# No germline mutations in supposed tumour suppressor genes *SAFB1 *and *SAFB2 *in familial breast cancer with linkage to 19p

**DOI:** 10.1186/1471-2350-9-108

**Published:** 2008-12-13

**Authors:** Annika Bergman, Frida Abel, Afrouz Behboudi, Maria Yhr, Jan Mattsson, Jan H Svensson, Per Karlsson, Margareta Nordling

**Affiliations:** 1 Department of Medical and Clinical genetics, Sahlgrenska Academy, Gothenburg, Sweden; 2 Department of Surgery, Sahlgrenska University hospital, Gothenburg, Sweden; 3 Department of Surgery, Skaraborg hospital, Skövde, Sweden; 4 Department of Oncology, Sahlgrenska University hospital, Gothenburg, Sweden

## Abstract

**Background:**

The scaffold attachment factor B1 and B2 genes, *SAFB1/SAFB2 *(both located on chromosome 19p13.3) have recently been suggested as tumour suppressor genes involved in breast cancer development. The assumption was based on functional properties of the two genes and loss of heterozygosity of intragenic markers in breast tumours further strengthened the postulated hypothesis. In addition, linkage studies in Swedish breast cancer families also indicate the presence of a susceptibility gene for breast cancer at the 19p locus. Somatic mutations in *SAFB1/SAFB2 *have been detected in breast tumours, but to our knowledge no studies on germline mutations have been reported. In this study we investigated the possible involvement of *SAFB1/SAFB2 *on familiar breast cancer by inherited mutations in either of the two genes.

**Results:**

Mutation analysis in families showing linkage to the *SAFB1/2 *locus was performed by DNA sequencing. The complete coding sequence of the two genes *SAFB1 *and *SAFB2 *was analyzed in germline DNA from 31 affected women. No missense or frameshift mutations were detected. One polymorphism was found in *SAFB1 *and eight polymorphisms were detected in *SAFB2*. MLPA-anlysis showed that both alleles of the two genes were preserved which excludes gene inactivation by large deletions.

**Conclusion:**

*SAFB1 *and *SAFB2 *are not likely to be causative of the hereditary breast cancer syndrome in west Swedish breast cancer families.

## Background

Breast cancer is the second most frequent cancer among women in the world. According to the Swedish Cancer Society 1,3 million women are estimated to develop breast cancer and the mortality rate was 36% in 2007. In 5–10% of all cases, an inherited susceptibility for breast cancer is predisposing women to develop the disease. Several genes have been identified as tumour suppressor genes that increase the overall risk to be affected by breast cancer. The two highly penetrant genes *BRCA1 *and *BRCA2 *are responsible for 25–40% of the familial cases depending on population. Other genes are causing rare cancer syndromes and are associated with an increased risk of breast cancer such as *TP53*, *PTEN*, *STK11/LKB1, TWIST1 *[1-4]. However, these genes have not yet proved causative in families with no other manifestations than breast cancer and there are probably other yet unknown tumour suppressor genes involved in breast tumorigenesis [5-7]. Large multi-center association studies have recently identified risk alleles of specific SNPs (single nucleotide polymorphism) as being associated with an increased risk of breast cancer [8,9]. A proportion of the familial cases may be explained by inheritance of several interacting low risk alleles. Nevertheless, there are multiple case families negative for *BRCA1 *and *BRCA2 *mutations with an inheritance mode that clearly appears as dominant and monogenic. In these families interacting low penetrant risk alleles therefore seem less likely to be the cause of the inherited predisposition. Recent linkage analyses performed by our group on Swedish families with hereditary breast cancer showed suggestive linkage to chromosome 10q, 12q and 19p [10]. In all, 74 individuals from 14 families, the majority originating from the west Swedish region, were genotyped using high density SNP microarrays. The identified linkage region at chromosome 19p (HLOD 2.1), overlapped with candidate regions identified by other groups and the region has been suggested to be the locus of a tumour suppressor gene [11,12]. The two genes, *SAFB1 *[Genbank NM_002967.2] and *SAFB2 *[Genbank NM_014649.2] are encoding scaffold attachment factor binding proteins that are localized in the nuclear matrix of the cell. Both genes are located at chromosome 19p13.3 and a complete loss of Safb1/2 protein has been reported in 20% of breast tumours [13]. The *SAFB1/2 *genes are coding for large proteins that have been characterized as proteins with multiple functions that in many ways are similar to functional properties of *BRCA1 *and *BRCA2*. Oesterreich and co-workers showed that Safb1 and Safb2-proteins function as transcriptional regulators mediated by repression of the oestrogen receptor which would point to a plausible role in carcinogenesis [14,15]. The group followed up this study by performing LOH analyses of 57 tumours (no reports on family history) with microsatellites in the 19p locus that harbours the *SAFB1/2 *genes [11]. They found a markedly high fraction of LOH in the marker D19S216 with 29 of 37 (78%) informative DNA samples with allelic deletion. Deletions in the same 19p region has also been reported in an earlier study by Lindblom et al. [12] in which 27% of the studied tumours (n = 82) showed LOH in this chromosomal region. We wanted to investigate whether *SAFB1 *and *SAFB2 *genes may be causative of hereditary breast cancer by analyzing patients with familiar breast cancer for inherited mutations in *SAFB1 *and *SAFB2*. To our knowledge, this is the first germline mutation analysis of the supposed tumour suppressor genes *SAFB1 *and *SAFB2*.

## Methods

### Patients

Germline DNA was extracted from venous blood, sampled from 31 affected women in 14 families with multiple cases of breast cancer and used as template in the germline mutation screening of the *SAFB1 *and the *SAFB2 *genes. Index cases have been analyzed and found negative for *BRCA1 *and *BRCA2 *mutations. Clinical characteristics such as age of onset, ovarian cancer occurrence etc. are presented in our previous publication [10]. Linkage analysis on affected women and relatives in the 14 families have previously shown positive linkage (HLOD 2.1) to the chromosomal region 19p13.3-q12, within which the *SAFB1 *and *SAFB2 *genes are located [10]. Two or three affected women from each family (n = 31) were included in the DNA sequence analyses of *SAFB1/2 *genes. One case from each family (n = 14) was included in the MLPA analysis. All patients in the study have given a written informed consent to participate in the study and the study was reviewed and approved by the University hospital ethic's committee, reference Ö-447-02.

### Polymerase chain reaction, PCR

The genomic structures of the *SAFB1 *and *SAFB2 *genes are very similar. The coding sequences of the two genes are distributed over 21 exons, spanning 45 kb and 36 kb respectively. The genes are ordered in a bidirectional, head to head state with a probable shared promoter [15]. *SAFB1 *and *SAFB2 *encode proteins with 915 and 953 amino acids respectively. The twenty-one exons (with flanking sequences) of *SAFB1 *and *SAFB2 *were amplified by PCR, primer sequences and PCR conditions are available in Additional file 1. PCR reactions were carried out in 20 μl volumes according to standard protocol. All reactions were run by touch-down PCR, in which the annealing temperature was gradually decreased during the ten first cycles to then continue the last 15 cycles at the lower annealing temperature.

### DNA sequencing

The PCR products were purified by magnetic beads (AMPure, Beckman-Coulter, ) and used as template in cycle sequencing reactions for analyses of sense and antisense strands. Diluted forward and reverse PCR primers were used as sequencing primers. The Big Dye Terminator chemistry 3.1 was used and reactions were carried out as recommended by the commercial supplier (Applied Biosystems, Foster city, USA). The sequence reaction products were analyzed on the Genetic Analyzer ABI3730 and the ABI3130 (Applied Biosystems). Sequencing analyses and comparisons to a reference sequence (*SAFB1 *NM_ 002967, *SAFB2 *NM_014649) was performed using the Seqscape software (Applied Biosystems).

### Multiplex ligation-dependent probe amplification, MLPA analysis

A screen for large deletions or amplifications over *SAFB1 *and *SAFB2* genes was performed by Multiplex Ligation-dependent Probe Amplification, MLPA. Five probes hybridizing to *SAFB1 *and two probes for *SAFB2 *were designed, see Table 1. The reference probe-mix P300 and reaction buffers were supplied by MRC-Holland (Amsterdam, NL) and the MLPA reactions were performed according to the manufacturer's recommendations. The amplified fragments were separated on an ABI 3130 Genetic Analyzer (Applied Biosystems) using Genescan-ROX 500 size standards (Applied Biosystems). Fragment analysis was performed with the Gene Mapper software (Applied Biosystems). Analysis of genomic deletions and duplications was based on the comparison of the peak areas of PCR-fragments generated from test samples and corresponding peak areas generated from control DNA samples. Normalizations of the peak areas were carried out according to the manufacturer's protocol.

**Table 1:** Hybridization sequences of MLPA probes.

**Probe**	**Gene/exon**	**Amplicon size (bp)**	Hybridization sequence LPO*	Hybridization sequence RPO**
1	*SAFB1 *exon 16	96	TACCATTCTGACTTTAACCGCCAGGACC	GCTTCCACGACTTTGACCACAGGGAC
2	*SAFB1 *exon 13	100	TCGAGGGACCGAACGGACTGTAGTAATGG	ATAAATCCAAAGGGGTGCCTGTGATTAGT
3	*SAFB2 *exon 4	112	GAGGACATGGAAGCAAGTCTGGAGAACCTGCAGAA	TATGGGCATGATGGACATGAGTGTGCTAGACGAAA
4	*SAFB1 *exon 10	116	TCACGATGTCCACAGCAGAAGAGGCCACAAAATGCAT	TAACCACCTGCACAAGACGGAGCTCCACGGAAAGATG
5	*SAFB2 *exon 21	120	GTCCCACTCGCTGCGAGTTTTCGGGTGGGCAGACGCACT	GTTGAATCTGGTAGCCAGGGTTCCCTCGAACTTGGGGGA
6	*SAFB1 *exon 14	124	GGATCGCAAATCAGCCAGCAGAGAGAAGCGGTCCGTCGTGT	CCTTTGATAAGGTCAAGGAGCCTCGGAAGTCAAGAGACTCA
7	*SAFB1 *exon 4	137	CCAGTCTGGAGAACTTGCAGGACATCGACATCATGGATATCAGTGTGT	TGGATGAAGCAGAAATTGATAATGGAAGCGTTGCAGATTGTGTCGAA

Amplicon size includes length of universal MLPA primer sequences.

*Left Probe Oligo ** Right Probe Oligo

## Results

**Figure 1:**
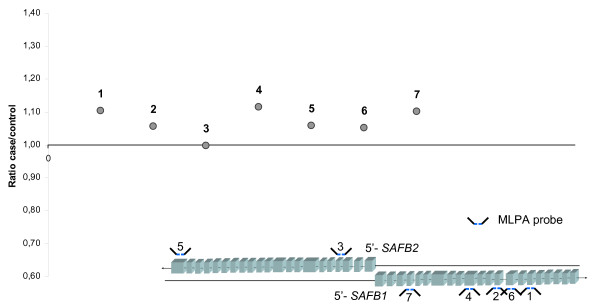
**The normalized values of peak areas for each probe's hybridization is given as the ratio of case/control, here shown for *SAFB *probes.** All probes have a case/ctrl ratio of approximately 1,0 which indicate that both alleles are preserved in the genome. Probe coverage in *SAFB1/2 *genes are shown below the graph, exon/intron sizes are not true to scale. Probe numbering refers to description in Table 1.

In order to investigate the potential role for *SAFB1/2 *as tumour suppressor genes involved in familial breast cancer, we performed a mutation screening of the two genes in families that display genetic linkage to the 19p locus. The complete coding sequence was analyzed by direct DNA sequencing. No frameshift or missense mutations were detected in any of the 31 analyzed DNA samples. One silent polymorphism was detected in the coding sequence of *SAFB1*. Three polymorphisms were detected in coding sequence of *SAFB2*, and further five polymorphisms in exon-flanking intronic sequence. All three coding polymorphisms were previously annotated in the SNP database, see Table 2. All sequence variations were analysed for aberrant splicing by submission of the altered sequence to the splice site prediction database BDGP (Berkely Drosophila Genome Project, ). One of the variants generated high score predictions of a novel splice donor site. The SNP was located in the 3' UTR-sequence, downstream of the termination codon, which makes it less likely that it affects the protein. As the allele does not segregate with disease we do not believe that the variant is pathogenic in a way that it would be affecting cancer susceptibility. Normalized ratios of case/control peak areas from the MPLA analysis showed no indications of deletions and the genes are not likely to be inactivated by entire gene deletions or by deletions of the targeted exons, see Fig. 1.

**Table 2:** Polymorphisms in SAFB1 and SAFB2 in 31 patients (62 alleles).

**Gene**	**Exon/Intron**	**Nucleotide position**	**Change**	**Frequency of minor allele**	**SNP annotation**
***SAFB1***	exon 8	c.1155	CCC>CCT Asp<Asp	1/62	NA*
***SAFB2***	exon 4	c.459	GAC>GAT Asp>Asp	1/62	NA*
	exon 9	c.1257	CGC>CGT Arg>Arg	3/62	rs806706
	intron 9	c.1296+31	T>C	1/62	NA*
	intron 13	c.1782+3	G>A	1/62	NA*
	intron 14	c.1919+18	A>G	42/62	rs10413286
	exon 16	c.2337	CAC>CAT His>His	4/62	NA*
	intron 17	c.2394+24	C>T	10/62	rs2285963
	3' UTR	c.2862+14	C>T	4/62	NA*

SNP annotations refer to NCBI SNPdb build 126.

*NA, Not Annotated.

## Discussion

Many genes have been suggested as genes predisposing for breast cancer. Since the discovery of *BRCA1 *and *BRCA2*, numerous genes have been associated with a moderate increase in risk and are thought to interact in a polygenic inheritance mode to increase the susceptibility for breast cancer. Attempts to identify further high penetrant genes such as *BRCA1 *and *BRCA2 *have not been successful and most of the research has now been directed towards identifying low risk alleles. However, there are families with multiple cases of breast cancer that present a pedigree with an undisputable dominant inheritance mode for which a polygenic model would not be appropriate. Several small and large linkage studies have failed to generate strong evidence for a susceptibility locus [16-18] and it seems likely that heterogeneity among families is a major obstacle when identifying genes with linkage studies.

Our previously reported linkage locus at 19p [10] overlaps with earlier identified chromosomal regions with frequent LOH in breast tumours [11,12]. Due to these findings the 19p-region appears as a reasonable candidate region for a tumour suppressor gene. One of the many functions attributed to *SAFB1 *and *SAFB2 *is that of a repressor of the estrogen receptor α, ERα, [13,15,19]. One could speculate that an inherited mutation in *SAFB1 *or *SAFB2 *followed by a somatic mutation later in life would lead to a complete gene inactivation and a disturbed regulation of ERα. Due to the many downstream targets of ER regulation [20], a lost repression of ERα would probably lead to an overexpression of a number of genes involved in growth and development. Somatic mutations of *SAFB1 *and *SAFB2 *have previously been observed in tumour DNA [11], but to our knowledge this is the first study of germline DNA from patients with hereditary breast cancer. Altogether, we identified eight polymorphisms in *SAFB2 *and one in *SAFB1*. None of the alterations caused an amino acid exchange which makes them less likely to be seriously affecting the functioning properties of the proteins. All but one alteration (*SAFB1 *exon 8) were found in regions with low degree of conservation which adds to the assumption that the variants have little or no effect on the resulting protein. Mutation analysis by DNA sequencing may fail to detect large genomic alterations such as entire or partial gene deletions. To address this issue we analysed the genes by MLPA analysis which is a suitable method for the detection of deletions or duplications. As no MLPA-probes were commercially available for the *SAFB1/2 *genes we developed and designed probes that hybridize to seven exons in *SAFB1 *and *SAFB2*. The probes used in the assay are spread over the genomic sequence of the two genes and an entire gene deletion or deletion of a targeted exon would have been detected as a reduction in peak area in the fragment analysis, see Fig 1.

The study has been restricted to mutation and deletion analysis of the coding sequence and there is of course the possibility of epigenetic or other less apparent alterations in the genes. There is also the risk of another tumour suppressor gene located in the close vicinity of *SAFB1 *and *SAFB2 *that would be the true basis for the observed linkage to the region. Another gene in the 19p-region that may appear as a suitable candidate is the *STK11/LKB1 *gene that is associated with the Peutz-Jehger's syndrome, in which breast cancer is a frequent manifestation. However, the hypothesis that the *STK11/LKB1 *gene is causative of familial breast cancer syndrome has already been tested by germline mutaion screening in Swedish breast cancer families [5]. As the families in that study were of similar origin as the families in the present study we find it unlikely that the *STK11/LKB1 *gene is the source of the 19p linkage.

## Conclusion

In conclusion, this study did not reveal any pathogenic germline mutations and no apparent genomic deletions in *SAFB1 *or *SAFB2*, and the hypothesis that the two genes would function as tumour suppressor genes could not be verified in this patient based material. The *SAFB1/2 *genes are not likely to confer a strong influence on familiar breast cancer in the west Swedish population.

## Competing interests

The authors declare that they have no competing interests.

## Authors' contributions

AB participated in design of the study and mutation analyses, coordinated the study, and drafted the manuscript. FA contributed to experiment design and manuscript revising. AfB contributed to study planning and revising the manuscript. MY contributed by developing experimental assays. JM contributed to collection of the patient material and revised the manuscript. JHS participated in collecting the patient material and revised the manuscript. PK participated in conceiving of the study and collecting patient material. MN participated in conceiving of the study, and participated in its design. All authors read and approved the final manuscript.

## Pre-publication history

The pre-publication history for this paper can be accessed here:



## Supplementary Material

Additional file 1 **PCR Primers and conditions.** The table shows all primer sequences used for the PCR reactions. Click here for file
